# Assessing the Training in Neurosurgery with the Implementation of VITOM-3D Exoscope: Learning Curve on Experimental Model in Neurosurgical Practice

**DOI:** 10.3390/brainsci13101409

**Published:** 2023-10-02

**Authors:** Giuseppe Roberto Giammalva, Federica Paolini, Flavia Meccio, Evier Andrea Giovannini, Alessandra Provenzano, Lapo Bonosi, Lara Brunasso, Roberta Costanzo, Rosa Maria Gerardi, Rina Di Bonaventura, Francesco Signorelli, Alessio Albanese, Domenico Gerardo Iacopino, Rosario Maugeri, Massimiliano Visocchi

**Affiliations:** 1 Neurosurgical Clinic, AOUP “Paolo Giaccone”, Post Graduate Residency Program in Neurologic Surgery, Department of Experimental Biomedicine and Clinical Neurosciences, School of Medicine, University of Palermo, 90127 Palermo, Italy; robertogiammalva@live.it (G.R.G.); federicapaolini94@gmail.com (F.P.); flavia.meccio@virgilio.it (F.M.); evierandreagiovannini@gmail.com (E.A.G.); alessandra.provenzano02@community.unipa.it (A.P.); lapo.bonosi@community.unipa.it (L.B.); roberta.costanzo@community.unipa.it (R.C.); rosamariagerardimd@gmail.com (R.M.G.); gerardoiacopino@gmail.com (D.G.I.); 2 Department of Neurosurgey, Fondazione Policlinico Universitario A. Gemelli IRCCS, Catholic University of the Sacred Heart, 00100 Rome, Italy; rina.dibonaventura@guest.policlinicogemelli.it (R.D.B.); francesco.signorelli@policlinicogemelli.it (F.S.); alessio.albanese@policlinicogemelli.it (A.A.); massimiliano.visocchi@policlinicogemelli.it (M.V.)

**Keywords:** exoscope, learning curve, operating microscope, VITOM, neurosurgery, training, neurosurgical practice

## Abstract

(1) Background: Innovation and continuous demand in the field of visual enhancing technologies and video streaming have led to the discovery of new systems capable of improving visualization and illumination of the surgical field. The exoscope was brought into neurosurgical routine, and nearly ten years later, modern 3D systems have been introduced and tested, giving encouraging results. (2) Methods: In order to evaluate the surgeon’s confidence with the exoscope and their increasing ability in terms of time spent and quality of the final achievement since their first encounter with the technique, an experimental trial on 18 neurosurgeons from a single Institution was performed to evaluate the learning curve for the use of the VITOM-3D exoscope in neurosurgical practice on a model of brain and dura mater. (3) Results: A significant improvement in the quality of the performance, number of errors made, and reduction in the time was found after the third iteration of the task, by when almost all the participants felt more comfortable and confident. No significant differences between senior neurosurgeons and resident neurosurgeons were reported. (4) Conclusions: Our results show that three iterations are enough to gain confidence with the exoscope from its first use, regardless of previous experience and training with an operating microscope.

## 1. Introduction

Adequate visualization of the surgical field is a primary need in order to perform safe and effective neurosurgical procedures. In particular, high-power illumination, depth of field, as well as the capability of tissue recognition and discrimination, are essential conditions to magnify the surgical anatomy in minimally invasive surgery. From its first introduction in 1960s, the operating microscope has benefited from several incremental technical improvements that enabled meeting the abovementioned requirements [1,2]. Hence, it gained a great popularity among neurosurgeons and it rapidly became the ‘gold standard’ visual enhancement technology in microsurgery. Operating microscopes have some drawbacks including poor mobility, accessibility, the large size of the instrument and poor ergonomics. The head of the microscope consists of binocular lenses, thus forcing the surgeons to maintain uncomfortable postures which may affect the concentration and so the surgical procedure. Moreover, when using the operating microscope, three-dimensional vision of the surgical field is limited to the first operator and his assistant, limiting a full participation of other members of the surgical team [3,4]. With the aim of overcoming these issues, engineering and innovation in the field of visual enhancing technologies and video streaming have led to the research of new systems capable of improving the visualization and the illumination of the surgical field [5,6]. These efforts finally gave birth to the (2D) extracorporeal telescope—or exoscope—whose first application in microsurgery was documented in 2010 [7]. Nearly ten years later, modern 3D systems have been introduced and tested, giving encouraging results [8]. As with all novel technology, the cutting-edge technical features of the exoscope clearly require a period of training for surgeons who approach this technology for the first time, aimed at improving their confidence with this optical tool. In order to evaluate the surgeon’s confidence with the 3D exoscope and their increasing ability in terms of time spent and quality of the final achievement since their first encounter with it, we designed an experimental training model of durotomy and duroplasty.

The primary aim of this study is to evaluate the learning curve in the use of a VITOM 3D exoscope (Karl Storz SE & Co. KG, Tuttlingen, Germany) for neurosurgical procedures since the first attempt at using this instrument. The secondary aim was to compare the results of the training with the years of surgical experience, to establish whether surgeons with greater experience have a shorter learning curve.

Our single center experience is presented, together with collected data, our learning curve model and report of participants’ perceptions.

## 2. Materials and Methods

At our Institution, since March 2021 the surgical room has been equipped with a VITOM 3D exoscope (Karl StorzSE & Co. KG, Tuttlingen, Germany). This system consists of a high-definition 4K stereoscopic camera, easily clamped on an auto-static holding arm (VERSACRANE, Karl Storz SE & Co. KG, Tuttlingen, Germany). The stereoscopic camera is linked to modules which receive and elaborate the video source (IMAGE1 S CONNECT and IMAGE1 S D3 Link, Karl Storz SE & Co. KG, Tuttlingen, Germany). The surgical field is enlightened by the high-definition camera itself through fiber optics connected to the light source (Power LED 300, Karl Storz SE & Co. KG, Tuttlingen, Germany). Optical parameters and video setting are controlled remotely through a remote controller (IMAGE1 Pilot, Karl Storz SE & Co. KG, Tuttlingen, Germany), which is mounted on an articulated arm and placed near the operator. Intraoperative stereoscopic high-definition images are displayed on a 32″ 3D 4K monitor, which is placed about 1.5 m in front of the operator. The learning curve was evaluated on a durotomy and duroplasty exercise using the VITOM-3D exoscope according to the experimental protocol described further below. The same protocol was repeated 6 consecutive times by each participant. For this study, 18 neurosurgeons from our Institution were enrolled. They were divided into two groups (“Junior” group and “Senior” group) according to their experience in neurosurgery. In particular, the “Junior” group was formed by resident neurosurgeons, whereas the “Senior” group was formed by consultant neurosurgeons.

### 2.1. Experimental Protocol

**Figure 1:**
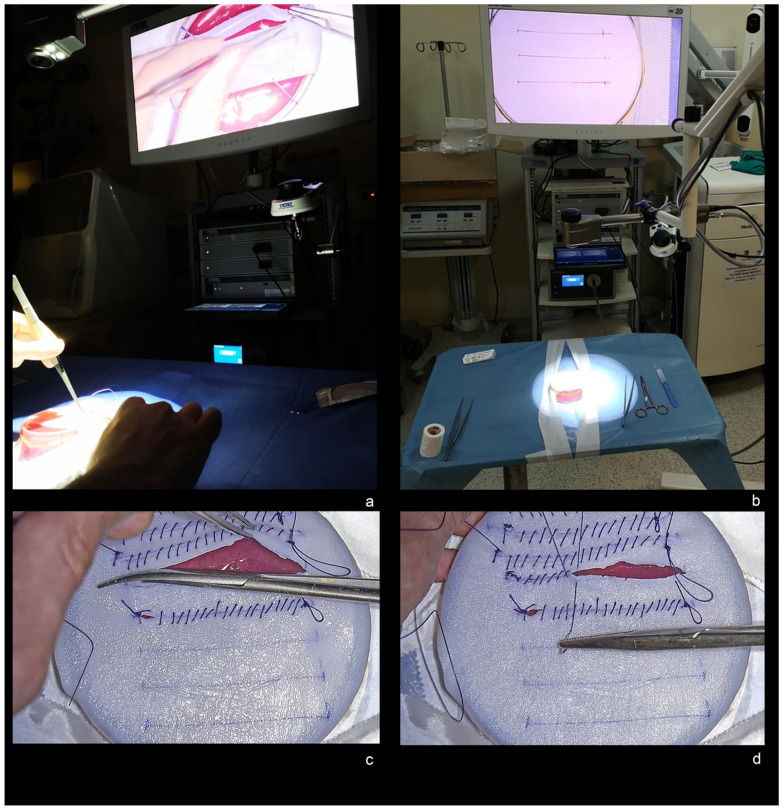
(**a**) Experimental model of brain and dura mater made of colored jelly capsule and covered by a dural substitute. (**b**) Exoscope set-up and calibration of the VITOM-3D at the beginning of the experimental task. (**c**) 5 cm durotomy. (**d**) Duroplasty with 3-0 Vycril continuous suture.

A model of brain and dura mater made of colored jelly capsule (mimicking brain) covered by a dural substitute (Durepair, Medtronic—Minneapolis, MN, USA) was used for the experimental protocol by each participant (Figure 1a).

The experimental protocol consisted of 3 tasks:

- Exoscope set-up (positioning of the VERSACRANE on surgical field and calibration of the VITOM-3D exoscope through focusing and white balancing) (Figure 1b);
- 5 cm durotomy (Figure 1c);
- Duroplasty using a 3-0 Vycril and continuous suture (Figure 1d).

Each task was timed from the evaluator’s START signal to the executor’s STOP signal at the end of the task. Participants were asked to perform the same protocol a total of 6 consecutive times.

### 2.2. Evaluation Score

The duroplasty task was evaluated using a 0-to-5-points scoring system according to the final result (Table S1). Errors were counted considering the number of *missed grips* (meant as the number of times when the participant failed to grip the dural substitute with surgical forceps), the number of *missed sutures* (meant as the number of times when the participant failed to reach the dural substitute with the needle), and the number of *jelly violations* (meant as the number of times when sutures went in error in to the jelly) by the same evaluator. A standardized questionnaire based on the Likert Scale was submitted to each participant at the end of each complete procedure in order to evaluate the perception of three domains: *self-perception of stress and quality of results*, *need for training in microscopy*, *overall comfort*.

Standard questions for the first domain (*self-perception of stress and quality of results*) were:

- How much do you rate the difficulty of the task? (0 = None; 5 = Very much)
- How much do you rate the stress during the task? (0 = None; 5 = Very much)
- How much do you rate the results of your performance? (0 = Very poor; 5 = Very good)
- How often do you think you made some mistakes? (0 = Never; 5 = Always)

Standard questions for the second domain (*need for training in microscopy*) were:

- How often did you need to convert in microscopy? (0 = Never; 5 = Always)
- How much training do you think is needed in order to obtain an optimal result? (0 = Never; 5 = Always)
- Can a former training in microscopy ensure an optimal result with these tasks in exoscopy? (0 = Strongly disagree; 5 = Strongly agree)
- May a further training in microscopy ensure an optimal result with these tasks in exoscopy? (0 = Strongly disagree; 5 = Strongly agree)

Standard questions for the third domain (*overall comfort*) were:

- How often did you have nausea? (0 = Never, 5 = Always)
- How often did you have ocular discomfort? (0 = Never, 5 = Always)
- How often did you have headache? (0 = Never, 5 = Always)
- How often did you have neck stiffness? (0 = Never, 5 = Always)

The results are reported in Table S2.

### 2.3. Statistical Analysis

Data were analyzed using Microsoft Excel (Microsoft Corp., Redmond, WA, USA), and statistical data were analyzed by analysis of variance (ANOVA) and linear regression test. *p*-value < 0.05 was considered statistically significant.

## 3. Results

The evaluation of the learning curve in the use of VITOM 3D exoscope in neurosurgical practice took place from April 2021 in a time-frame of four months. For the purpose of this study, 18 neurosurgeons were enrolled and divided into two groups; the “Junior group” was formed by 13 resident neurosurgeons from their 1st to their 4th post-graduate year; the “Senior group” was formed by five consultant neurosurgeons with a mean 11.4 ± 5.12 years of neurosurgical experience each (range 6 to 20 years).

### 3.1. Timing

**Figure 2:**
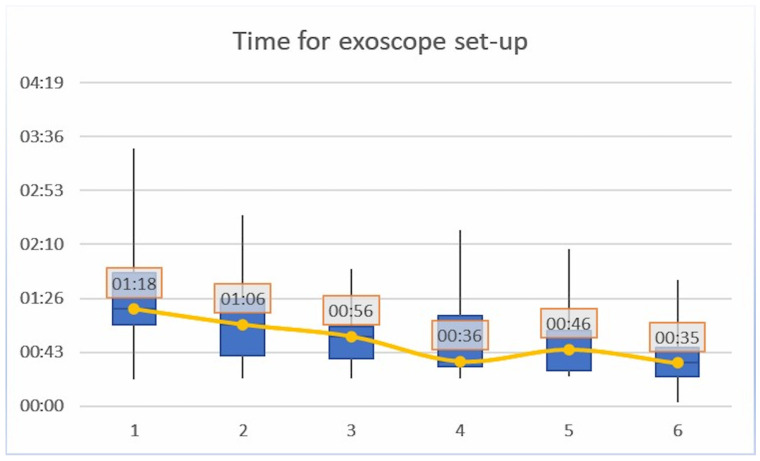
Timing for exoscope set-up; a mean progressive reduction was shown for each consecutive iteration of the task (*p* < 0.01, R = 0.45).

Regarding the timing for *exoscope set-up*, a mean progressive reduction was shown for each consecutive iteration of the task (*p* < 0.01, R = 0.45). In particular, a median time of 01′18″ (±00′49″) was recorded for the iteration #1; 01′06″ (±00′36″) for iteration #2; 00′56″ (±00′27″) for iteration #3; 00′36″ (±00′31″) for iteration #4; 00′46″ (±00′28″) for iteration #5; and 00′35″ (±00′22″) for iteration #6 (Table 1). The time curve flattened after the iteration #3 as shown in Figure 2. No significant differences were recorded between the Junior and Senior groups.

Regarding the timing for *durotomy*, a mean progressive reduction was shown for each consecutive iteration of the task (*p* < 0.05, R = 0.24). In particular, a median time of 01′00″ (±01′26″) was recorded for the iteration #1; 01′08″ (±00′57″) for iteration #2; 00′57″ (±01′05″) for iteration #3; 00′37″ (±01′24″) for iteration #4; 00′30″ (±00′51″) for iteration #5; and 00′26″ (±00′39″) for iteration #6 (Table 2). The time curve showed a progressive reduction without significant difference between Junior and Senior groups, although lower overall mean time which was recorded for the Senior group.

**Figure 3:**
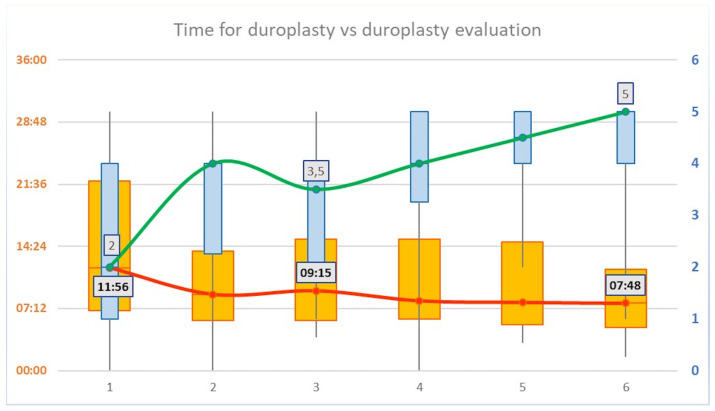
Timing vs. evaluation score for duroplasty. A mean progressive reduction in the time for duroplasty was shown for each consecutive iteration of the task (*p* < 0.05, R = 0.2, values on the left Y axis). Duroplasty evaluation score showed a mean progressive increase for each consecutive iteration of the task (*p* < 0.01, R = 0.42, values on the right Y axis). An inversion of the trend is shown at the third iteration of the task, both for the time and for the evaluation of duroplasty.

Regarding the timing for *duroplasty*, a mean progressive reduction was shown for each consecutive iteration of the task (*p* < 0.05, R = 0.2). In particular, a median time of 11′56″ (±08′31″) was recorded for the iteration #1; 08′50″ (±07′03″) for iteration #2; 09′15″ (±05′41″) for iteration #3; 08′06″ (±07′15″) for iteration #4; 07′55″ (±06′32″) for iteration #5; and 07′48″ (±05′13″) for iteration #6 (Table 3). The time curve flattened after the iteration #3 as shown in Figure 3. No significant differences in the time curve were recorded between Junior and Senior groups, although lower overall mean time was recorded for the Senior group.

### 3.2. Evaluation

The final result for the duroplasty task was evaluated with a 0-to-5 point scale, according to the completion, the tightness, the symmetry and integrity of the duroplasty; 5 points were assigned for the best results, whereas 0 points were assigned in the case of incomplete duroplasty with multiple jelly violations.

The duroplasty evaluation score showed a mean progressive increase for each consecutive iteration of the task (*p* < 0.01, R = 0.42). In particular, a median score of 2 ± 1.67 was reported for the iteration #1; a median score of 4 ± 1.56 was reported for the iteration #2; a median score of 3.5 ± 1.25 was reported for the iteration #3; a median score of 4 ± 1.37 was reported for the iteration #4; a median score of 4.5 ± 0.98 was reported for the iteration #5; and a median score of 5 ± 1.1 was reported for the iteration #6 (Table 4). After a slight decrease, the evaluation curve showed a constant increase after the iteration #3 as shown in Figure 3. No significant differences in the curve were recorded between Junior and Senior groups.

**Figure 4:**
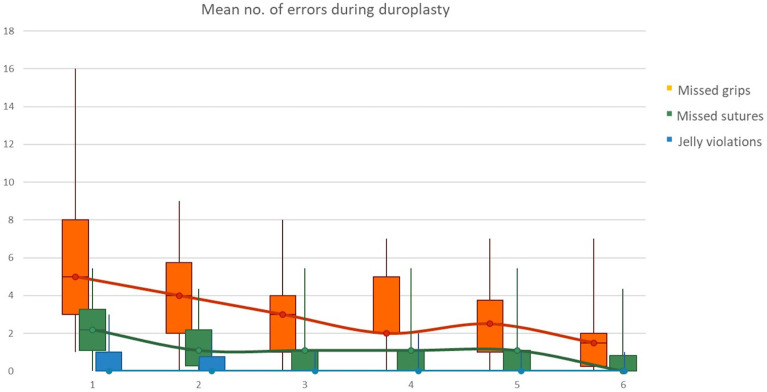
Mean number of encountered errors during duroplasty; a constant and progressive reduction was recorded for *missed grips*, *missed sutures* and *jelly violations.*

Regarding encountered errors during duroplasty, a constant and progressive reduction was recorded not only for *missed grips*, but also for *missed sutures* and *jelly violations* for each consecutive iteration of the task as shown in Figure 4.

### 3.3. Self-Assessment

**Figure 5:**
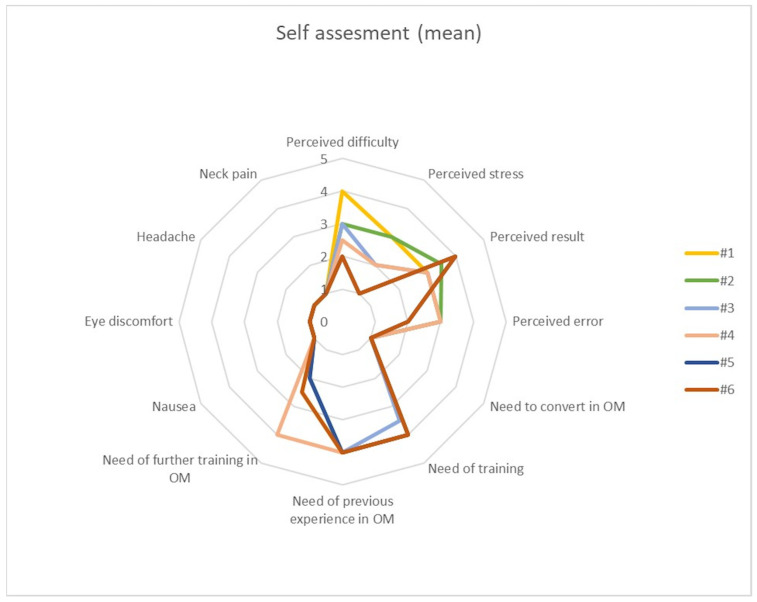
Mean self-assessment score for each of the six iterations of each task, comprising three domains: *self-perception of stress and quality of results*, *need for training in microscopy*, *overall comfort.*

Self-assessment took the form of three domains for consideration: self-perception of stress and quality of results, need for training in microscopy, overall comfort according to the abovementioned standardized questionnaire. Regarding the first domain (self-perception of stress and quality of results), every participant reported a lower perceived stress and difficulty and better perceived results for each iteration at the end of experimental protocol. Regarding the second domain (need for training in microscopy), no one needed to convert the task in microscopy and the self-reported need to undertake further training in microscopy decreased with every subsequent iteration of the tasks. On the other hand, it was a common perception that previous training in microscopy and experience with an operative microscope would have aided the execution of the experimental protocol in exoscopy. Notably, a training in exoscopy was constantly considered fundamental for improvement in performance. Regarding the third domain (overall comfort), no one complained about physical discomfort during the whole experimental protocol. No differences in self-assessment were noticed between Junior and Senior groups. Results are summarized in Figure 5.

## 4. Discussion

The possibility of enhancing the visualization of the surgical field is of outmost importance in neurosurgical practice. Since the introduction of magnifier glasses, the visualization and magnification of the surgical field have constantly improved thanks to the use of the operating microscope, endoscope and exoscope. Since its recent introduction, it has been shown that the 3D exoscope guarantees a clear view of the surgical field at least equal to that of operating microscopes, with some additional technical improvements.

Our Institution was equipped with the VITOM-3D exoscope in March 2021. As soon as this equipment arrived, we conducted an experimental trial to evaluate the learning curve for the use of the VITOM-3D exoscope in daily neurosurgical practice on a model of brain and dura mater. The aim was to study the learning curve of neurosurgeons with different years of surgical practice on their approaching new instrumentations and technologies. None of them had ever used the exoscope before this experiment.

This trial involved thirteen resident and five senior neurosurgeons, who were first asked to set up the VITOM-3D exoscope. Next, they were asked to perform six times the same task of durotomy and duroplasty with a watertight suture on a realistic and easily reproducible jelly model of brain and dura-mater. At the end of these tasks, the evaluator administered the participant a self-assessment questionnaire in order to evaluate participant skills and familiarity with using the exoscope, their self-perception of their own results and the rate of their stress and discomfort in facing the task with the aid of a novel instrument used for the first time. The results of this experimental trial demonstrated that all the participants showed a significant improvement in terms of quality of performance, number of occurrences of errors, and reduction in the time taken for the task after its third iteration. Since senior neurosurgeons will have used an operating microscope for a long time and may be more confident with it than resident neurosurgeons, this experimental protocol aimed also to highlight potential differences between these two groups in their use of a 3D exoscope for the first time. However, our results showed no significant differences between senior neurosurgeons and resident neurosurgeons. Almost all the participants felt more comfortable and confident with this technique after the third iteration, and no discomfort or side effects were experienced throughout the training protocol. Furthermore, almost all the participants reported that using the 3D exoscope was as practical as the operating microscope; however, it was a common perception that previous experience with the operating microscope could have been helpful in improving the maneuverability of and ease of use of the 3D exoscope for the first time. Moreover, no differences in the morphology of the learning curve were shown between senior and resident neurosurgeons. Adaptation to the exoscope is considered a limitation in different studies; however, it could be easier for neurosurgeons with previous experience in endoscopic surgery in view of the similar working position [2]. From the results of our study, no limitations or difficulties were highlighted from neurosurgeons who were trained in the use of operating microscopes, albeit our senior group was small. Moreover, learning curves were comparable between the two groups.

Although a preliminary monocentric experiment with a small sample size, we believe that this study could represent the starting point for creating a more extensive and easily repeatable training protocol, thus improving the learning curve for the utilization of the exoscope in the daily practice of neurosurgery.

In the literature, several studies can be found that were conducted to compare the use of VITOM-3D with the operating microscope. Both instruments have notably been widely compared regarding their advantages and disadvantages during surgery [9,10,11,12]. In particular, the exoscope camera has a wider range of motion and requires much less space compared to the operating microscope. Given the smaller size of the exoscope, shifting from microscopic to macroscopic vision can be achieved without moving the exoscope; moreover, intraoperative space-occupying devices such as neuronavigation and neurophysiological monitoring devices can be used without moving the exoscope, thus maintaining the microscopic view of the surgical field. Furthermore, due to its larger depth of field and longer focal distance, the exoscope moves effortlessly, suspended above the surgical field, providing illumination, image quality and three-dimensional visualization at least equivalent to those of the operating microscope [12,13]. In addition, surgeons are able to maintain a more comfortable position of the neck during the procedure since images are visualized on a large monitor. In fact, according to recent studies, the operator’s fatigue using an exoscope is reduced compared to when operating a microscope; however, it remains unclear if this could influence the clinical outcome [4]. Accordingly, the need to convert from exoscope to operating microscope during a surgical procedure has been reported in selected cases [10]. Moreover, VITOM-3D may require more frequent refocusing and rebalancing than an operating microscope according to some authors [4,12].

In a comprehensive study led by Calloni et al., an in-depth exploration was undertaken to evaluate the learning curve and perspectives of resident neurosurgeons when employing two distinct visualization devices: an operative microscope (Leica OHX, Leica Biosystems, Wetzlar, Germany) and a 3D exoscope (Orbeye, Olympus, Tokyo, Japan). According to their results, there was no statistically significant difference in the time required for resident neurosurgeons to identify the first structure using either the microscope or the exoscope. However, significant improvements in speed were noted with both devices in subsequent attempts, underscoring the adaptive nature of the resident neurosurgeons’ learning curve. Interestingly, the exoscope demonstrated a significant advantage in terms of speed from the first to the third iterations, when compared to the microscope. The study also showed that initially, the more experienced resident neurosurgeons performed better with the exoscope. Overall, resident neurosurgeons favored the exoscope, citing its user-friendliness and intuitive nature, thus suggesting its potential as a future standard in visualization devices [13].

As regards training and education, the use of the exoscope also gives several opportunities for application in education, as it allows a wider fruition and better involvement of all the team within the surgical room, thus overcoming the intrinsic limits of operating microscopes whose direct view is restricted to only two operators [12,14].

Notwithstanding its advantages, such cutting-edge technical features of the exoscope clearly require a period of training for surgeons who approach this technology for the first time, with a view to improving their skills with this optical tool. Indeed, an effective use of the exoscope requires reverse vision and maneuvering skills, since monitors are typically far from the hands of the operator [3,7,15].

In this regard, Silva et al. tracked the progress of two expert neurosurgeons, with substantial backgrounds in microneurosurgery using conventional operating microscopes, as they adopted the use of an exoscope over the course of a year. During this year, they engaged in a training program involving a complex dissection task on a chicken wing model. Their progress was assessed in two aspects: exoscope handling and fine motor skills under high magnification. Noteworthy findings from this investigation included a consistent reduction in dissection time, despite increased manipulation of the exoscope, indicating a steep learning curve. Hand and instrument movements also exhibited significant transformations. As the participants gained confidence, superfluous movements decreased, and they embraced more efficient microsurgical techniques. The study further highlighted variables affecting dissection time, encompassing focus-related issues, unnecessary movements, and zooming behavior, thereby emphasizing the profound impact of skills and experience in exoscope-assisted surgery [16].

From our perspective, our results showed that three iterations are enough to gain confidence with the exoscope from its first use, regardless of previous experience and training with an operating microscope.

Our study presents some limitations: in particular, the small sample size, the lack of numerical uniformity between the two groups and the use of an experimental model of brain and dura mater. However, the lack of numerical uniformity between groups is due to the small number of senior neurosurgeons available at our Institution compared to resident neurosurgeons. Moreover, the use of the experimental model and the lack of in vivo application could also be considered an advantage since they standardize the task and the assessment of the learning curve, eliminating the bias that would accompany the application of a given task on different patients and surgical conditions.

We consider that our study sheds light on the use of this optical technology in neurosurgery and promotes the exposure of young trainees to the use of the 3D exoscope in view of the fast learning curve and the rapid increase in confidence with this device soon after its first use. However, further studies are needed in order to assess the use of the 3D exoscope and the learning curve in vivo, in different surgical scenarios and with different group of trainees.

## 5. Conclusions

The 3D exoscope has been recently introduced in neurosurgery and requires a period of training for surgeons who approach this technology for the first time. At our Institution, we conducted the first experimental trial to evaluate the learning curve for the use of the VITOM-3D exoscope in daily neurosurgical practice on a model of brain and dura mater. The results of this experimental trial demonstrated that all the participants showed a significative improvement in performing the experimental task using the 3D exoscope. Our results notably showed that three iterations are enough to gain confidence with the exoscope from its first use, regardless of previous experience and training with the operating microscope. Moreover, no differences in the morphology of the learning curve were shown between senior neurosurgeons and young trainees.

## Figures and Tables

**Table 1:** General characteristics of each participant. Yrs Of NS: number of years of experience in neurosurgery.

Group	Subject	Yrs of NS	Gender	Age
Junior	1	0	M	25
	2	0	F	27
	3	0	F	25
	4	0	M	26
	5	0	M	29
	6	1	M	32
	7	1	F	29
	8	2	M	28
	9	3	F	27
	10	1	F	26
	11	3	F	28
	12	3	F	27
	13	2	M	28
Senior	14	6	M	37
	15	7	F	33
	16	10	F	36
	17	14	M	40
	18	20	M	45

**Table 2:** Timing for exoscope set-up. Yrs of NS: number of years of experience in neurosurgery. #: number of task’s iteration. St Dev: standard deviation.

Group	Subject	Yrs of NS	#1	#2	#3	#4	#5	#6
Junior	1	0	00:02:43	00:02:05	00:01:05	00:01:16	00:00:58	00:00:47
	2	0	00:02:54	00:00:49	00:01:00	00:01:34	00:00:55	00:00:49
	3	0	00:01:10	00:01:26	00:01:33	00:02:21	00:00:25	00:00:39
	4	0	00:01:51	00:00:57	00:01:00	00:01:30	00:00:53	00:00:26
	5	0	00:01:00	00:00:38	00:00:54	00:01:02	00:01:01	00:00:37
	6	1	00:03:27	00:01:34	00:00:59	00:01:17	00:01:05	00:00:46
	7	1	00:01:22	00:02:33	00:01:36	00:00:35	00:01:55	00:01:13
	8	2	00:01:30	00:02:18	00:01:44	00:00:59	00:02:06	00:01:41
	9	3	00:00:21	00:00:22	00:00:24	00:00:33	00:00:26	00:00:21
	10	1	00:00:48	00:00:57	00:01:50	00:00:37	00:00:28	00:00:37
	11	3	00:02:50	00:01:18	00:00:48	00:00:35	00:00:24	00:00:23
	12	3	00:01:06	00:00:32	00:00:37	00:00:23	00:00:35	00:00:03
	13	2	00:01:35	00:01:14	00:00:58	00:00:38	00:00:52	00:00:59
Senior	14	6	00:01:14	00:00:34	00:00:23	00:00:30	00:00:31	00:00:20
	15	7	00:00:57	00:01:02	00:00:33	00:00:22	00:01:02	00:00:30
	16	10	00:01:09	00:01:25	00:00:40	00:00:31	00:00:28	00:00:22
	17	14	00:01:22	00:01:09	00:00:48	00:00:33	00:00:38	00:00:28
	18	20	00:01:05	00:00:34	00:00:22	00:00:24	00:00:39	00:00:32
Mean			00:01:18	00:01:06	00:00:56	00:00:36	00:00:46	00:00:35
St Dev			00:00:49	00:00:36	00:00:27	00:00:31	00:00:28	00:00:22

**Table 3:** Timing for durotomy. Yrs of NS: number of years of experience in neurosurgery. #: number of task’s iteration.

Group	Subject	Yrs of NS	#1	#2	#3	#4	#5	#6
Junior	1	0	00:01:37	00:01:55	00:01:42	00:06:08	00:02:24	00:01:35
	2	0	00:03:36	00:02:05	00:01:04	00:02:53	00:03:09	00:01:46
	3	0	00:00:50	00:01:23	00:03:46	00:01:28	00:00:51	00:00:54
	4	0	00:02:04	00:01:17	00:01:32	00:00:41	00:00:21	00:00:14
	5	0	00:04:56	00:02:15	00:01:54	00:00:44	00:00:56	00:01:38
	6	1	00:03:26	00:03:54	00:03:36	00:02:15	00:01:52	00:01:21
	7	1	00:04:18	00:01:46	00:03:00	00:01:21	00:00:54	00:02:14
	8	2	00:02:34	00:02:17	00:01:47	00:01:23	00:02:02	00:01:25
	9	3	00:00:23	00:00:22	00:00:21	00:00:29	00:00:22	00:00:21
	10	1	00:01:00	00:00:53	00:00:34	00:00:22	00:00:28	00:00:26
	11	3	00:00:39	00:00:19	00:00:11	00:00:13	00:00:11	00:00:09
	12	3	00:01:35	00:00:59	00:00:51	00:00:46	00:00:29	00:00:27
	13	2	00:01:01	00:00:43	00:00:38	00:00:33	00:00:41	00:00:29
Senior	14	6	00:00:13	00:00:10	00:00:09	00:00:08	00:00:11	00:00:10
	15	7	00:00:49	00:01:25	00:00:56	00:00:31	00:00:32	00:00:26
	16	10	00:00:08	00:00:09	00:00:59	00:00:19	00:00:08	00:00:08
	17	14	00:00:30	00:00:34	00:00:37	00:00:22	00:00:22	00:00:21
	18	20	00:00:37	00:00:25	00:00:19	00:00:24	00:00:24	00:00:18
Mean			00:01:00	00:01:08	00:00:57	00:00:37	00:00:30	00:00:26
St Dev			00:01:26	00:00:57	00:01:05	00:01:24	00:00:51	00:00:39

**Table 4:** Timing for duroplasty. Yrs of NS: number of years of experience in neurosurgery. #: number of task’s iteration.

Group	Subject	Yrs of NS	#1	#2	#3	#4	#5	#6
Junior	1	0	00:28:03	00:24:47	00:22:40	00:27:02	00:25:31	00:12:24
	2	0	00:23:18	00:17:42	00:16:15	00:20:03	00:22:11	00:21:30
	3	0	00:25:29	00:14:02	00:17:22	00:24:40	00:17:28	00:17:43
	4	0	00:31:16	00:12:54	00:10:49	00:13:36	00:13:16	00:09:48
	5	0	00:18:26	00:30:02	00:12:00	00:15:26	00:15:15	00:12:32
	6	1	00:13:51	00:11:05	00:11:05	00:14:41	00:13:44	00:09:40
	7	1	00:23:11	00:15:36	00:16:33	00:12:22	00:08:58	00:08:25
	8	2	00:18:01	00:13:23	00:21:05	00:21:16	00:18:33	00:16:12
	9	3	00:09:09	00:07:15	00:08:07	00:07:27	00:06:16	00:05:00
	10	1	00:06:18	00:07:41	00:08:30	00:07:30	00:08:13	00:06:29
	11	3	00:10:00	00:10:00	00:10:01	00:08:41	00:07:36	00:07:11
	12	3	00:06:47	00:05:42	00:06:06	00:05:56	00:04:58	00:05:46
	13	2	00:18:07	00:06:01	00:06:28	00:07:00	00:06:38	00:08:31
Senior	14	6	00:04:19	00:04:02	00:03:51	00:02:35	00:03:15	00:01:35
	15	7	00:09:12	00:06:51	00:05:44	00:06:14	00:06:43	00:05:09
	16	10	00:07:23	00:05:00	00:04:26	00:03:46	00:04:07	00:03:03
	17	14	00:05:59	00:05:06	00:05:41	00:05:22	00:04:23	00:04:35
	18	20	00:04:38	00:04:24	00:04:58	00:05:30	00:05:01	00:04:36
Mean			00:11:56	00:08:50	00:09:15	00:08:06	00:07:55	00:07:48
St Dev			00:08:31	00:07:03	00:05:41	00:07:15	00:06:32	00:05:13

## Data Availability

Not applicable.

## Supplementary Materials

The following supporting information can be downloaded at: https://www.mdpi.com/article/10.3390/brainsci13101409/s1, Table S1: Duroplasty evaluation. Yrs of NS: number of years of experience in neurosurgery. #: number of task’s iteration; Table S2: Mean score of self-assessment for the whole cohort of participants. #: number of task’s iteration.

## References

[1] Byvaltsev V.A., Akshulakov S.K., Polkin R.A., Ochkal S.V., Stepanov I.A., Makhambetov Y.T., Kerimbayev T.T., Staren M., Belykh E., Preul M.C. (2018). Microvascular Anastomosis Training in Neurosurgery: A Review. Minim. Invasive Surg..

[2] Pafitanis G., Hadjiandreou M., Alamri A., Uff C., Walsh D., Myers S. (2020). The Exoscope versus Operating Microscope in Microvascular Surgery: A Simulation Non-Inferiority Trial. Arch. Plast. Surg..

[3] Fiani B., Jarrah R., Griepp D.W., Adukuzhiyil J. (2021). The Role of 3D Exoscope Systems in Neurosurgery: An Optical Innovation. Cureus.

[4] Oertel J.M., Burkhardt B.W. (2017). Vitom-3D for Exoscopic Neurosurgery: Initial Experience in Cranial and Spinal Procedures. World Neurosurg..

[5] Maugeri R., Giammalva R.G., Iacopino D.G. (2016). On the Shoulders of Giants, with a Smartphone: Periscope in Neurosurgery. World Neurosurg..

[6] Visocchi M., Mattogno P., Ciappetta P., Barbagallo G., Signorelli F. (2020). Combined Transoral Exoscope and OArm-Assisted Approach for Craniovertebral Junction Surgery: Light and Shadows in Single-Center Experience with Improving Technologies. J. Craniovertebr. Junction Spine.

[7] Mamelak A.N., Nobuto T., Berci G. (2010). Initial Clinical Experience with a High-Definition Exoscope System for Microneurosurgery. Neurosurgery.

[8] Visocchi M., Mattogno P.P., Signorelli F. (2021). Exoscope and OArm: What We Can Learn in Craniovertebral Junction Surgery. J. Neurosurg. Sci..

[9] Angileri F.F., Esposito F., Scibilia A., Priola S.M., Raffa G., Germanò A. (2019). Exoscope-Guided (VITOM 3D) Single-Stage Removal of Supratentorial Cavernous Angioma and Hemangioblastoma: 3-Dimensional Operative Video. Oper. Neurosurg..

[10] Burkhardt B.W., Csokonay A., Oertel J.M. (2020). 3D-Exoscopic Visualization Using the VITOM-3D in Cranial and Spinal Neurosurgery. What Are the Limitations?. Clin. Neurol. Neurosurg..

[11] Rossini Z., Cardia A., Milani D., Lasio G.B., Fornari M., D’Angelo V. (2017). VITOM 3D: Preliminary Experience in Cranial Surgery. World Neurosurg..

[12] Yoon W.S., Lho H.W., Chung D.S. (2021). Evaluation of 3-Dimensional Exoscopes in Brain Tumor Surgery. J. Korean Neurosurg. Soc..

[13] Calloni T., Antolini L., Roumy L.G., Nicolosi F., Carrabba G.G., Di Cristofori A., Fon-tanella M.M., Giussani C.G. (2023). Exoscope and operative microscope for training in micro-neurosurgery: A laboratory investigation on a model of cranial approach. Front. Surg..

[14] Beez T., Munoz-Bendix C., Beseoglu K., Steiger H.-J., Ahmadi S.A. (2018). First Clinical Applications of a High-Definition Three-Dimensional Exoscope in Pediatric Neurosurgery. Cureus.

[15] Birch K., Drazin D., Black K.L., Williams J., Berci G., Mamelak A.N. (2014). Clinical Experience with a High Definition Exoscope System for Surgery of Pineal Region Lesions. J. Clin. Neurosci..

[16] Silva J.M., Rustemi O., Vezirska D.I., Niemelä M., Lehecka M., Hafez A. (2023). Taming the exoscope: A one-year prospective laboratory training study. Acta Neurochir. (Wien).

