# Supplementary material for: Assessing the Training in Neurosurgery with the Implementation of VITOM-3D Exoscope: Learning Curve on Experimental Model in Neurosurgical Practice
Source: Brain Sci. 2023 Oct 2;13(10):1409. doi: 10.3390/brainsci13101409 (PMC10605262; doi:10.3390/brainsci13101409)
Supplement: Supplementary file 1 [file brainsci-13-01409-s001.zip › brainsci-2588963-supplementary.pdf]

Table S1. Duroplasty evaluation. Yrs of NS: number of years of experience in neurosurgery. #: number of task's iteration.

Group	Subject	Yrs of NS	#1	#2	#3	#4	#5	#6
Junior	1	0	1	4	2	2	4	4
	2	0	1	0	5	0	4	5
	3	0	5	4	2	4	4	5
	4	0	0	4	2	2	2	2
	5	0	1	1	2	4	5	5
	6	1	1	4	4	5	3	4
	7	1	2	5	1	4	5	4
	8	2	0	2	2	2	2	1
	9	3	4	4	5	5	5	5
	10	1	4	5	4	5	4	4
	11	3	2	4	4	4	5	5
	12	3	5	5	3	5	5	5
	13	2	1	1	3	4	5	4
Senior	14	6	4	3	4	5	4	4
	15	7	4	4	5	4	5	5
	16	10	1	1	3	3	4	5
	17	14	2	4	4	5	5	5
	18	20	4	5	5	4	5	5
Mean			2	4	3,5	4	4,5	5
St Dev			1.67	1.56	1.25	1.37	0.98	1.1

Table S2. Mean score of self-assessment for the whole cohort of participants. #: number of task's iteration.

Domain	Self-assessment	#1	#2	#3	#4	#5	#6
Self-perception of stress and quality of results	Perceived difficulty	4	3	3	2,5	2	2
	Perceived stress	3	3	2	2	1	1
	Perceived result	3	3,5	3	3	4	4
	Perceived error	3	3	3	3	2	2
Need for training in microscopy	Need to convert in OM	1	1	1	1	1	1
	Need for training	4	4	3,5	4	4	4
	Need for previous experience in OM	4	4	4	4	4	4
	Need for further training in OM	2,5	2	2	4	2	2,5
Overall comfort	Nausea	1	1	1	1	1	1
	Eye discomfort	1	1	1	1	1	1
	Headache	1	1	1	1	1	1
	Neck pain	1	1	1	1	1	1
